# Efficient high-risk human papillomavirus screening through pooled testing: a simulation study based on real-world data in low- and middle-income countries

**DOI:** 10.7189/jogh.16.04275

**Published:** 2026-07-24

**Authors:** Fengming Pan, Marcel JW Greuter, Jaap AR Koot, Heather Cubie, Naheed Nazrul, Carolyn Nakisige, Jogchum Beltman, Geertruida H de Bock, Jurjen van der Schans

**Affiliations:** 1 Department of Epidemiology, University Medical Centre Groningen, University of Groningen, Groningen, the Netherlands; 2 Department of Radiology, University Medical Centre Groningen, University of Groningen, Groningen, the Netherlands; 3 Unit of Global Health, Department of Health Sciences, University Medical Centre Groningen, Groningen, the Netherlands; 4 Global Health Academy, University of Edinburgh, Edinburgh, UK; 5 Health Sector, Friendship NGO, Dhaka, Bangladesh; 6 Department of Gynaecologic-Oncology, Uganda Cancer Institute, Kampala, Uganda; 7 Department of Gynaecology, Leiden University Medical Centre, Leiden University, Leiden, the Netherlands; 8 Department of Economics, Econometrics and Finance, University of Groningen, Groningen, the Netherlands; 9 Faculty of Management Sciences, Open University, Heerlen, the Netherlands

**Keywords:** human papillomavirus DNA tests, pooled testing, sample pooling, cervical cancer, screening, low- and middle-income countries

## Abstract

**Background:**

The World Health Organization recommends high-risk human papillomavirus (hrHPV) testing as the primary method for cervical cancer screening. Combining samples in a pool can reduce the expense associated with large numbers of commercially available tests. This study simulated the potential test-saving impact of pooled hrHPV testing using real-world data from two low- and middle-income countries (LMICs).

**Methods:**

We simulated a two- and three-stage hrHPV pooling in Bangladesh (hrHPV prevalence 2.10%) and Uganda (hrHPV prevalence 22.52%) using real-world laboratory data. Measurements included the number of tests per stage, the total number of tests, and the percentage of tests saved. We performed sensitivity analysis to assess the impact of a 1.00% loss of sensitivity and specificity per additional sample in the pool on the false-negative rate (FNR).

**Results:**

In Bangladesh, the two-stage strategy (eight samples per pool) and the three-stage strategy saved 71.21% and 77.21% of tests, respectively, compared to testing individually. In Uganda, the two-stage strategy (three samples per pool) reduced the number of tests by 15.34% compared to testing individually. Assuming loss of sensitivity and specificity due to pooling, the FNR was higher (FNR = 0.012–0.134) when hrHPV prevalence was low under the two-stage strategy.

**Conclusions:**

Pooled hrHPV testing has the potential to reduce the number of tests used and enhance screening capacity in resource-limited settings. Notably, a lower prevalence of hrHPV allows for more efficient pooled testing. Despite the potential loss of sensitivity and specificity, pooled hrHPV testing could offer benefits for LMICs and warrant further verification.

Cervical cancer is the fourth most frequently diagnosed cancer and the fourth leading cause of cancer death in women globally [1], the World Health Organization (WHO) estimating that the age-standardised rate of incidence and mortality are 14.1 and 7.1 per 100,000 women per year worldwide in 2022 [2]. The incidence and mortality of cervical cancer are highest in low- and middle-income countries (LMICs), reflecting significant inequalities due to a lack of cervical cancer screening and human papillomavirus (HPV) vaccination [1]. In Bangladesh, cervical cancer is the second most predominant cancer among women, with age-standardised incidence and mortality rates of 11.3 and 7.0 per 100,000 women per year, respectively [3]. In Uganda, the incidence and mortality of cervical cancer are among the highest worldwide [4], with age-standardised incidence and mortality rates of 53.8 and 40.6 per 100,000 women [5].

Untreated high-risk HPV (hrHPV) infection causes almost all cervical cancers [6]. The WHO recommend hrHPV testing as the primary method of cervical cancer screening [7]. However, the commercially available HPV-DNA tests that can perform the genotyping recommended by WHO are expensive and require advanced and costly laboratory equipment and training [8,9]. Scaling up the implementation of hrHPV testing to national screening will significantly increase the costs of consumables, such as test kits. Options are needed to make hrHPV testing more affordable in LMICs while retaining efficiency. One possibility is to adopt pooled testing strategies that combine samples from multiple individuals and test them as a single pool rather than testing each sample individually [10]. During the COVID-19 pandemic, pooling strategies were widely used for SARS-CoV-2 detection, including in LMICs [11–13]. The approach has also been advocated for detecting hepatitis B and C viruses [14,15], HIV [16], and *Neisseria gonorrhoeae* [17].

In large-scale screening or when testing resources are limited, pooled testing can improve detection capacity, help estimate prevalence, save reagents, reduce costs, ease pressure on healthcare services, and provide timely information to clinicians and patients [18]. If the pooled test result is negative, all included samples are regarded as negative. If positive, at least one sample in this pool must be positive, and the samples are tested again, either individually or in smaller pools. Although several pooled testing strategies are available [19,20], most rely on mathematical simulations of idealised scenarios or on a fixed number of samples to validate a theory. A better understanding is needed of the effectiveness of pooling strategies for hrHPV testing in real-world LMIC settings, including the impact of unpredictable sample volumes and the potential loss of sensitivity and specificity due to pooling. In this study, we aimed to assess the real-world potential for test-saving with hrHPV pooling strategies in two LMICs with different hrHPV prevalences.

## METHODS

### Context

We sourced the data from the Prevention and Screening Innovation Project towards Elimination of Cervical Cancer (PRESCRIP-TEC) [21], which focused on increasing the adoption of cervical cancer screening in resource-poor or hard-to-reach settings in Bangladesh, India, Uganda, and Slovakia. In Bangladesh and Uganda, cervical cancer screening relies on hrHPV self-sampling tests as the primary screening tool. For positive hrHPV tests, women are invited for follow-up examination by visual inspection with acetic acid to identify lesions, and if this was positive, eligible women were treated with thermal ablation or referred if cervical cancer was suspected. Participating laboratories use a real-time polymerase chain reaction (PCR) assay (GeneXpert; Cepheid, Sunnyvale, California, USA) to detect hrHPV DNA (HPV16, 18, 45, 31, 33, 35, 52, 58, 51, 59, 39, 56, 66, 68) based on a non-batch, random-access platform [22,23].

The project received ethical approval from the Institutional Review Board of the International Centre for Diarrheal Disease Research in Bangladesh, the Uganda Cancer Institute Research Ethics Committee, and the Uganda National Council for Science and Technology. Participants signed an informed consent form before taking part in the study.

### Study design

In this simulation study, we used a resampling method to model the implementation of pooled hrHPV testing in real-world settings, aiming to assess the benefit and feasibility of this approach. The hrHPV testing dates and results from the two laboratories were used to create country-specific data sets. To assess the effect of irregularities in daily testing volume on pooling efficiency, we assumed that all samples collected were pooled daily. It was also assumed that no loss of sensitivity or specificity occurred due to the pooling strategy in the base analysis because this has not been shown in any study to date.

### Pooling strategies

We evaluated two- and three-stage pooling strategies. In the two-stage pooling strategy, 19 scenarios were simulated by pooling two to 20 samples per pool. If the total number of tests per day was less than the specified pooling number, we combined all tests into a single pool for testing; if multiple pools were formed per day, tests were combined into another pool when the remaining tests were fewer than that number. If the pooled result was negative, no follow-up testing was required. If the pooled result was positive, the second stage was to test each sample in the pool individually.

The second pooling strategy used a modified three-stage approach, which has been shown to reduce the number of tests compared to other methods [19]. Considering the laboratory conditions and the level of operational skill in LMICs, this approach enabled further exploration of reducing the number of tests. As described in the literature [19], this method provides a mathematical framework to determine the optimal pooling strategy under ideal conditions, including the number of testing stages and the pool size at each stage, given a specified pathogen prevalence. And this method restricts the number of testing stages to a maximum of three to ensure practical feasibility in laboratory settings.

Following the pre-estimation of pathogen prevalence, the number of testing stages and the pool size at each stage are determined using the formula – the number of testing stages:



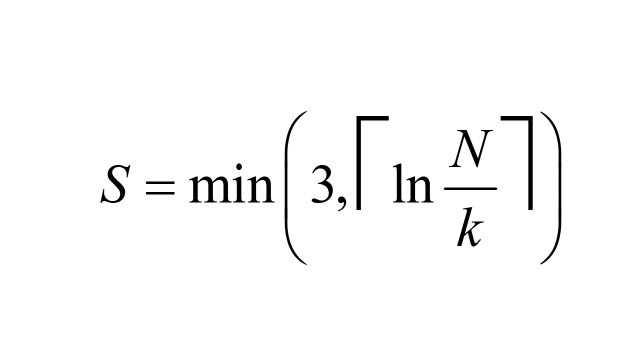



The pool size in each stage:



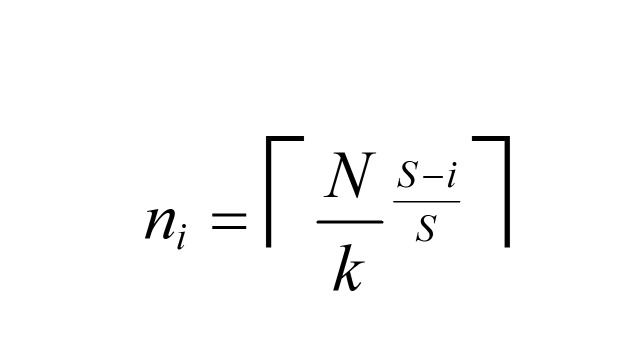



where *n_i_* is the pool size in stage *i*, *S* is the number of stages, *N* is the total number of tests, *k* is the number of positive samples, the ceiling function ⌈·⌉ is used to round up the pool size, ensuring it is practical for real-world implementation.

### Sensitivity analysis

Due to the lack of relevant studies on loss of hrHPV testing performance after pooling, expert opinions from a consultant clinical virologist and two principal investigators from PRESCRIP-TEC in Bangladesh and Uganda were sought to estimate this loss. This informed the need to perform sensitivity analyses assuming a 1% decrease in sensitivity and specificity for each additional sample in the pool, as a hypothetical assumption for exploratory modelling. We then calculated the percentage of tests saved and the false-negative rate (FNR), which represents missed diagnoses, to explore the impact of the performance loss on the pooling strategies. Sensitivity and specificity referred to the ability of the hrHPV test to detect the hrHPV. The FNR was defined as the proportion of truly positive samples incorrectly classified as negative (Methods S1 in the **Online Supplementary Document**).

Additionally, to account for potential variations in operational workflows, sensitivity analyses were conducted under the base-case scenario, assuming no loss in sensitivity or specificity after pooling, exploring alternative batching strategies, including two-day and seven-day pooling intervals, which may reflect multi-day batching or sample transport from decentralised collection sites.

### Statistical analysis

The pooled testing results were calculated using different pooling strategies. Outcome indicators included the number of tests at each stage, the total number of tests, and the percentage of tests saved in the multi-stage strategy.



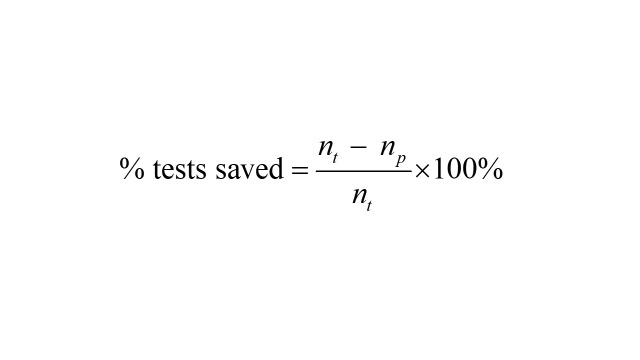



where *n_t_* is the total number of tests if tested individually, and *n_p_* is the total number of tests if pooled. To mitigate sampling randomness, the results were averaged over 1000 simulations per scenario, and 95% confidence intervals (CIs) were calculated where applicable. For metrics involving test counts, values were ceiling-rounded to the nearest integer, as fractional counts are not interpretable. The optimal pool size was defined as the pool size that yielded the lowest average total number of tests across 1000 simulations. We performed simulations and statistical analyses using *R*, version 4.5.2 (R Foundation for Statistical Computing, Vienna, Austria).

## RESULTS

In Bangladesh, 7,828 hrHPV tests were performed in 337 days and 164 (2.10%) were positive. The daily test volume was mostly regular, with a median (Mdn) of 21 and an interquartile range (IQR) of 17. In Uganda, 6,625 sample tests were performed in 67 days, and 1,492 (22.52%) were positive. The daily test volume was more irregular in this case (Mdn = 43; IQR = 123).

### Design of the three-stage strategy

Based on the prevalence of hrHPV in the Bangladesh data set and the formula described in the method [19], the optimal strategy was to divide the pooled testing into three stages: in stage one, samples were grouped into pools of 14 for testing; in stage two, samples were retested in pools of four after removing the negative pools; in stage three, samples in any positive pools were tested individually to identify positive samples. By contrast, the Ugandan data set had a higher hrHPV prevalence, which, according to the formula [19], yielded an optimal two-stage strategy: in stage one, samples were pooled in groups of three for testing; in stage two, positive pools were resolved by testing each individual sample. Since this approach aligned with the existing two-stage strategy, the three-stage strategy was not applied to the Uganda data set.

### The Bangladesh data set

**Figure 1:**
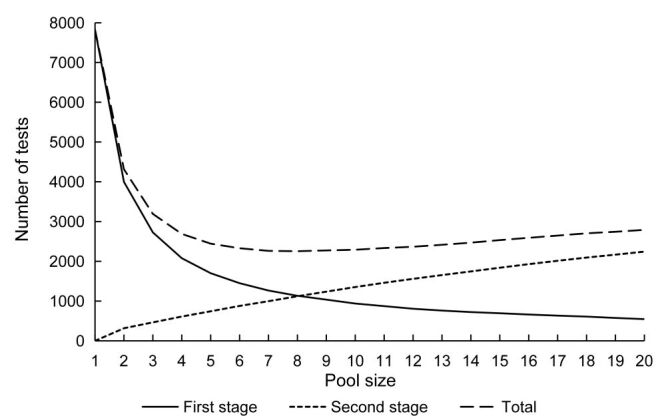
Bangladesh pooled test simulation of hrHPV. First stage of the two-stage strategy. Second stage of the two-stage strategy.

Using the two-stage approach, the number of tests in the first stage (pooled stage) decreased, and the number of tests in the second stage (individual stage) increased as the pool size increased (2–20 samples per batch) (**Figure 1**). As the pool size increased, the total number of tests first declined rapidly, then levelled off. The optimal pool size, yielding the lowest total number of tests, was eight samples per pool, with an estimated mean of 2,254 tests (95% CI = 2,252–2,256), saving 71.21% (95% CI = 71.18–71.23) of tests compared to individual testing.

The three-stage strategy required an average of 1,784 tests (95% CI = 1,783–1,785), with stages one (n = 723), two (n = 480), and three (n = 581) tests. The number of tests could be further reduced by 20.85% compared to the two-stage strategy, and by 77.21% compared to individual testing.

### The Uganda data set

**Figure 2:**
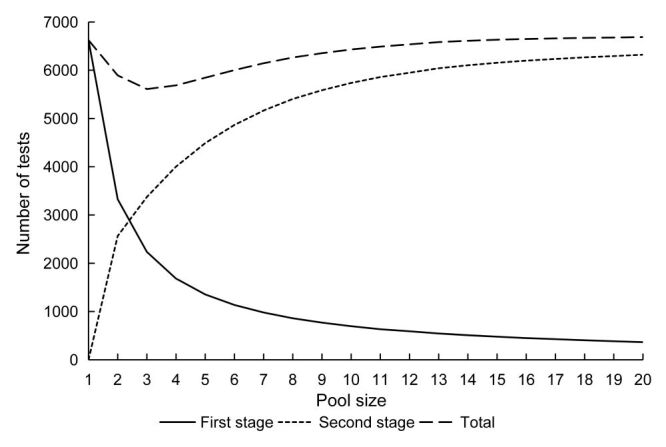
Pooling testing simulation results of the Uganda laboratory data set. First stage of the two-stage strategy. Second stage of the two-stage strategy.

Given the higher hrHPV prevalence in the Uganda data set, the total number of tests in the two-stage strategy had a U-shaped curve (**Figure 2**). Three samples per pool yielded the fewest total tests, saving 15.34% (95% CI = 15.31–15.37) compared with individual testing. When the pool size exceeded 15, the total number of tests required for pooled testing (n = 6,633; 95% CI = 6,630–6,636) was higher than for individual testing (n = 6,625).

### Sensitivity analysis

Sensitivity analyses were conducted assuming a 1.00% decrease in the sensitivity and specificity of hrHPV testing for each additional sample in a pool (**Table 1**). The two-stage strategy saved more tests in the low hrHPV prevalence condition in Bangladesh than in the high hrHPV prevalence condition in Uganda, but the FNR was also higher. In Bangladesh, the optimal pool size was reduced to seven from eight in the base scenario, where the loss in hrHPV performance was not considered. The highest percentage of tests saved decreased by 4.26%, while the FNR increased progressively as the pool size grew. The FNR at 20 samples per pool was 11.17 times higher than at two samples per pool. In the three-stage strategy used with the Bangladesh data set, pool sizes in the first and second stages were 14 and four, respectively; this saved 75.87% (95% CI = 75.83–75.89) of the tests with a 1.34% reduction compared to the base scenario. However, the FNR was 0.140, which was higher than when the two-stage strategy had 20 samples per pool.

**Table 1:** Results of the sensitivity analysis of two pooling strategies

Pool size	Sensitivity and specificity (%)	Bangladesh data set		Uganda data set	
		Tests saved, % (95% CI)*	**FNR**	Tests saved, % (95% CI)*	**FNR**
Two-stage strategy					
*2*	99	44.00 (43.98–44.00)	0.012	10.84 (10.82–10.87)	0.009
*3*	98	57.52 (57.50–57.54)	0.024	15.35 (15.31–15.38)	0.015
*4*	97	63.22 (63.20–63.23)	0.030	14.76 (14.72–14.81)	0.021
*5*	96	65.67 (65.64–65.70)	0.037	13.22 (13.16–13.27)	0.024
*6*	95	66.65 (66.62–66.68)	0.049	11.73 (11.67–11.79)	0.027
*7*	94	66.95 (66.90–66.99)	0.055	10.64 (10.58–10.72)	0.030
*8*	93	66.61 (66.56–66.65)	0.061	9.90 (9.83–9.98)	0.032
*9*	92	65.99 (65.93–66.04)	0.067	9.42 (9.34–9.49)	0.033
*10*	91	65.44 (65.38–65.51)	0.079	9.48 (9.40–9.57)	0.035
*11*	90	64.63 (64.55–64.69)	0.085	9.68 (9.58–9.75)	0.036
*12*	89	63.92 (63.85–64.00)	0.091	9.98 (9.89–10.07)	0.037
*13*	88	63.07 (62.99–63.15)	0.098	10.46 (10.37–10.55)	0.038
*14*	87	62.26 (62.17–62.34)	0.104	11.08 (10.97–11.17)	0.038
*15*	86	61.39 (61.29–61.48)	0.104	11.77 (11.67–11.88)	0.039
*16*	85	60.60 (60.51–60.71)	0.110	12.47 (12.35–12.57)	0.039
*17*	84	59.75 (59.64–59.85)	0.116	13.36 (13.24–13.48)	0.040
*18*	83	58.88 (58.78–58.99)	0.122	14.16 (14.04–14.28)	0.040
*19*	82	58.21 (58.11–58.33)	0.128	15.00 (14.87–15.12)	0.040
*20*	81	57.84 (57.73–57.96)	0.134	15.95 (15.82–16.08)	0.040
Three-stage strategy					
*14/4†*	87/97	75.87 (75.83–75.89)	0.140		

CI – confidence interval, FNR – false-negative rate

*CIs were reported for outcomes with observable variability across simulations.

†14/4 indicates that the pool sizes in the first and second stages are 14 and four, respectively.

In the Uganda data set, using the two-stage pooling strategy, the percentage of tests saved was 15.35% with a pool size of three. Although there was a 2% loss in sensitivity and specificity for the pooled hrHPV test, there was no reduction in the number of tests saved compared to the base scenario. The percentage of tests saved at 20 samples per pool exceeded that at three samples per pool, but the FNR was 2.67 times greater.

The percentage of tests saved varied slightly as pooling intervals increased, and the optimal pool size under the two-stage strategy remained unchanged (Figures S1 and S2 in the **Online Supplementary Document**).

## DISCUSSION

Our results indicate that pooled hrHPV testing could be an effective strategy to reduce screening costs in resource-limited settings. By combining multiple samples into pools, the number of tests required can be significantly reduced without compromising accuracy, provided the pool sizes are carefully chosen. Assuming that the loss of sensitivity and specificity due to pooling affects the FNR, the benefit of pooling will decrease as prevalence increases. While these findings highlight the potential of pooled testing, this study focuses on test reduction rather than a comprehensive evaluation of cost-effectiveness or implementation of pooled testing. Future studies should evaluate the real-world feasibility, cost-effectiveness, and implementation challenges of pooled hrHPV testing in diverse settings.

A study investigating pooled testing of SARS-CoV-2 with a background prevalence of 2.4% showed that four-sample pooled testing resulted in 66% and 59% reductions in resource use and turnaround times, respectively [24]. When using a four-sample pooled testing in the two-stage strategy in Bangladesh, a similar 65.65% reduction in hrHPV tests used was achieved, but this increased to 71.21% with an optimum eight samples per pool. Furthermore, using the three-stage strategy with pool sizes of 14 in the first stage and four in the second stage, the number of tests saved was further increased to 77.21% compared to individual testing. In a study of SARS-CoV-2 with a 2% prevalence, a similar three-stage pooling approach achieved a 77% reduction in the number of tests, using pool sizes of 20 and five for the first and second stages, respectively [25].

The prevalence of hrHPV is the main factor influencing the efficiency of pooled testing. A higher percentage of tests was saved when hrHPV prevalence was low, because positive samples had a lower probability of being present in pools, allowing rapid exclusion of negative samples. In Bangladesh, where hrHPV prevalence is low, one hrHPV test purchased within the PRESCRIP-TEC project cost about USD 20 as of 2022 [26]. Implementing a two-stage pooling strategy with a pool size of eight may reduce test kit costs by approximately USD 1.42 million per 100,000 women screened, based on test savings and excluding additional operational or programme costs. For LMICs with an unknown HPV prevalence, introducing hrHPV testing should be a stepwise process. Since pooled testing involves potential challenges, it would be more appropriate to consider after laboratories have gained sufficient experience with individual hrHPV testing and can ensure consistent quality and accuracy. As part of this process, they will obtain a rough estimate of the local hrHPV prevalence to guide the next step.

An irregular daily testing volume means that each pooling sample does not necessarily reach the specified size. Ideally, enough samples are obtained and the performance of hrHPV testing does not change after pooling. Using the two-stage strategy with a pool size of eight could save 72.2% of the tests used when the prevalence is 2.1%, as calculated by the Robert Dorfman method [10]. However, when applying this pooling strategy to a data set simulating daily detection, the percentage of tests saved decreases slightly. This is because, in low-prevalence settings, some pools will not reach the specified sizes, and detection efficiency will be reduced. With a higher prevalence of 22.5%, pooling three samples together could save 13.2% of tests [10]. However, an irregular daily testing volume will improve detection efficiency, possibly because there is a higher probability of having only one sample per pool, thereby avoiding duplicate testing in the second stage.

Of note, the pool size that saved the most tests when simulating the two-stage strategy aligned with calculations from the Robert Dorfman method [10], suggesting that daily variations in test volume did not affect the optimal pool size. Using the Bangladesh data set, we observed that the total number of tests did not change significantly once the pool size exceeded four, suggesting that in settings with low hrHPV prevalence, the number of samples per pool had less impact on efficiency after reaching a certain size. This provides considerable flexibility when determining the pool size.

Loss of test performance due to pooling is a major concern, but little has been reported on the sensitivity and specificity of pooled hrHPV testing. This study assumed a 1.00% decrease in sensitivity and specificity for each additional sample added to the pool to simulate dilution effects under real-world conditions. This showed that multi-stage strategies and larger pool sizes are associated with a higher risk of missed diagnoses despite improving test throughput. One study examining the performance of pooling longitudinally collected cervical specimens for hrHPV detection found similar trends and recommended that pool sizes not exceed five samples [27]. Similar observations have been made in studies of pooled testing for SARS-CoV-2 [18,28,29], emphasising the need for conservative pool sizes to balance efficiency gains against diagnostic accuracy. In addition, the potential clinical implications of missed hrHPV infections should be carefully considered. Missed hrHPV infections may prevent individuals from entering triage pathways, thereby delaying further diagnostic evaluation and potential treatment, particularly in settings with limited opportunities for repeat screening. However, in many low- and middle-income settings where access to screening remains limited, pooled testing may be a pragmatic approach to achieve broader screening coverage when individual testing would otherwise be unaffordable or unavailable.

Inaccuracies may arise from low viral loads, poor swab quality, insufficient sample volumes, or DNA degradation during sample processing, all of which may be exacerbated by pooling [20]. As pool sizes increase, samples with low viral loads, particularly those near the limit of detection, are more likely to yield false negatives. Optimising real-time PCR protocols, such as using additional amplification cycles, can improve the detection of low viral load samples and avoid missed diagnoses [24,25]. Moreover, a study reported approximately 5% of hrHPV specimens were suspected false positives after pooling, indicating inherent variability in assay performance [27]. Further studies on the actual reductions in the sensitivity and specificity of HPV testing after pooling are needed in LMICs. Given international variations in HPV testing methods, kits, and laboratory machines, each healthcare setting should validate their pooling strategies independently [30].

Time is a crucial factor in pooled testing [31]. Considering the variability in laboratory capacity and staff expertise in low- and middle-income settings, the extent to which turnaround time may be shortened or delayed is difficult to quantify. Therefore, this factor was not incorporated into the model in this study. Although samples can be stored at room temperature for up to one week [32], participants should receive results promptly to prevent disease progression. This study assumed that samples collected within one day would be processed the following day to facilitate pooling. However, the manufacturer states that test quality is not affected by a few days’ delay, so it may be possible to combine samples taken on different days. Laboratories can plan pooled testing schedules based on throughput and available human resources. If the analysis of a particular batch of samples might be significantly delayed, pooling should not be used for that batch. Furthermore, long-term declines in hrHPV prevalence due to increased coverage for HPV vaccination and cervical cancer screening may necessitate periodic adjustments of pool sizes based on previous test positivity rates [33].

Although pooled testing can increase efficiency compared to individual testing, it adds complexity to sample preparation and tracking, increases the potential for contamination, requires additional training for laboratory technicians in LMICs, and poses significant logistical challenges in implementing theoretically optimised pooling designs [34]. The complexity in workflows can be mitigated by using appropriate tracking software and standardised operating procedures to minimise the risk of errors or sample mix-ups [34]. Nonetheless, the additional costs and burdens associated with laboratory management of the pooling process must be considered in future research.

Several low-cost HPV assays are currently under development [35–38]. For example, the novel Zebra BioDome version of the ScreenFire HPV assay not only reduces testing costs (approximately USD 6 per test for scale-up) but also streamlines the HPV testing process and reduces the risk of laboratory contamination [35]. This provides a source of optimism for hrHPV testing in LMICs. The development of lower-cost HPV tests coupled with pooled testing has the potential to greatly accelerate the coverage of cervical cancer screening and assist in achieving the WHO ‘90-70-90’ targets for vaccination-screening-treatment by 2030 [39].

This is the first study using real-world data from LMICs to explore the potential of pooled hrHPV testing on test saving. It offers valuable insights about improving hrHPV testing efficiency, expanding coverage of cervical cancer screening, and promoting healthcare equity. The present study also has some limitations. Although approximately 7.00% of the data in the Uganda data set lacked an hrHPV testing date, the prevalence in the remaining records was consistent with the original data, suggesting limited impact on our findings. Due to the limited availability of national screening population-based data, the hrHPV prevalence estimates were derived from PRESCRIP-TEC project, which may have introduced selection bias for the prevalence estimates. Our model also simplified real-world HPV testing scenarios and did not account for ambiguous test results potentially overestimating data provided on the number of tests saved. In addition, contamination risk, clerical errors, throughput bottlenecks, assay performance after pooling, and diagnostic impacts remain uncertain and require further evaluation.

## CONCLUSIONS

Pooled hrHPV testing may represent a pragmatic approach to enhance screening capacity in resource-limited LMIC settings. Although irregular daily testing volumes can affect pooling effectiveness, countries with a low hrHPV prevalence, such as Bangladesh, could achieve meaningful benefits with the appropriate pooling strategy. Once hrHPV testing is established in the national screening strategies of LMICs, pooled testing could be an attractive option for cost-effective implementation, especially when combined with lower-cost testing methods.

## Additional material


Online Supplementary Document

## Data Availability

**Data availability:** The model code files used in this analysis are available on DataverseNL (https://doi.org/10.34894/LO4AA6), shared as part of the PRESCRIP-TEC project. Files relevant to this analysis have been labelled accordingly in their descriptions.
